# Supplementary material for: Degradation bottlenecks and resource competition in transiently and stably engineered mammalian cells
Source: Nat Commun. 2025 Jan 2;16:328. doi: 10.1038/s41467-024-55311-w (PMC11696530; doi:10.1038/s41467-024-55311-w)
Supplement: Supplementary file 1 — Supplementary information [file 41467_2024_55311_MOESM1_ESM.pdf]

Supplementary Information

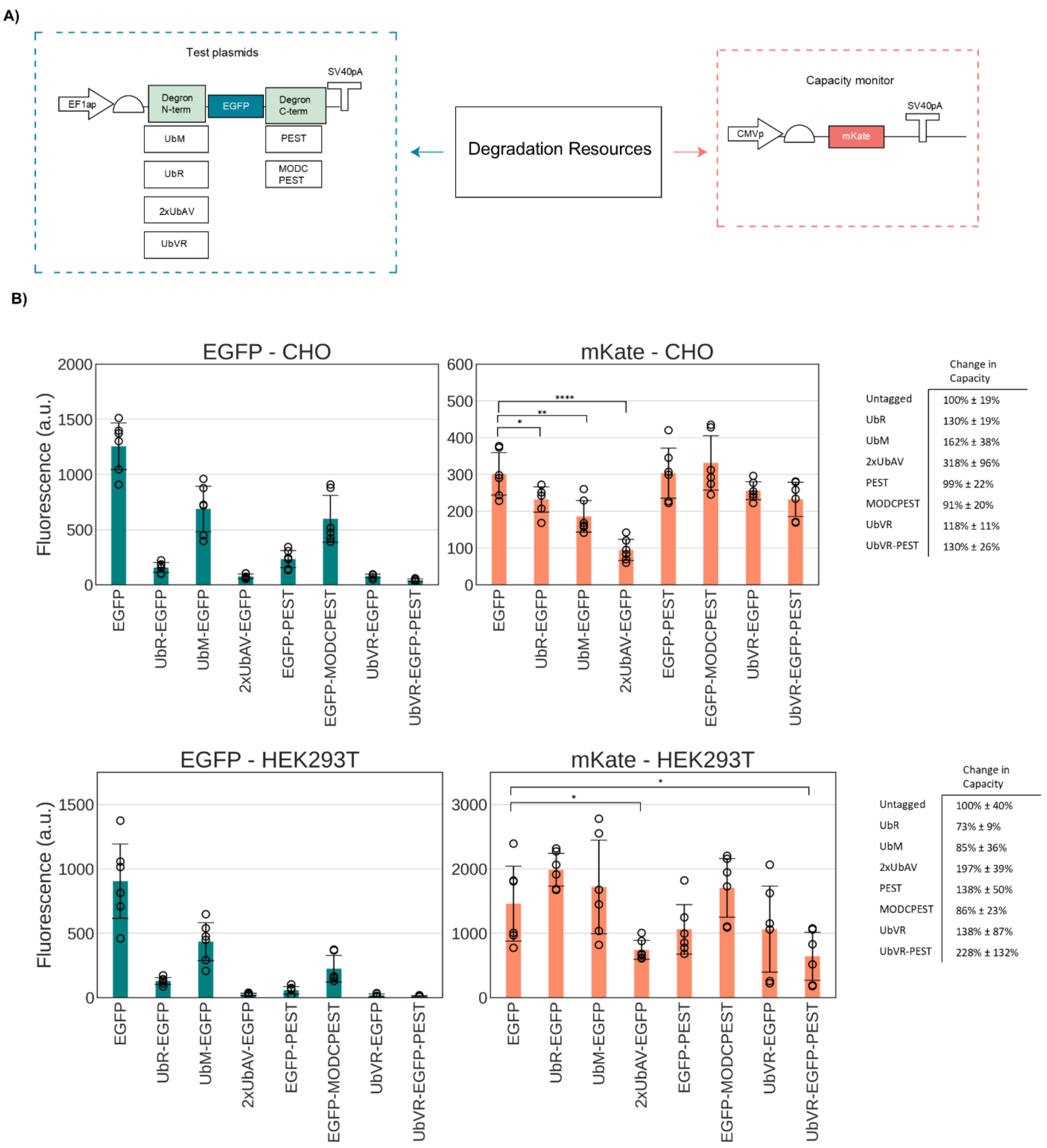

Figure S1. An untagged mKate monitor results in the absence of competition for degradation. A) Schematics of the genetic construct library where the untagged mKate monitor was co-transfected with seven EGFP-expressing test constructs tagged with UbM, UbR, 2xUbAV, UbVR, PEST or MODCPEST in both HEK293T and CHO-K1. **B)** Flow cytometry data for the untagged monitor in CHO-K1 (top) and HEK293T (bottom). Test plasmid and capacity monitor fluorescence levels are reported as mean fluorescence (arbitrary units) ± std. Data derived from three independent experiments each with two biological repeats. A two-tailed Student T-Test was used, where P values are denoted as follows: *****<0.00005, ****<0.0005, ***<0.0005, **<0.005, *<0.05, and it was annotated only on the mKate charts with reference to the control, EGFP versus capacity monitor, for clarity purposes. Tables report 1/(mKate of EGFP-tagged condition/mKate of EGFP untagged condition) to quantify change in degradation capacity upon adding the degron ± the ratio of the standard deviation to the mKate mean multiplied by change in capacity. The number of biological repeats for each sample and exact P values are reported in Source data file.

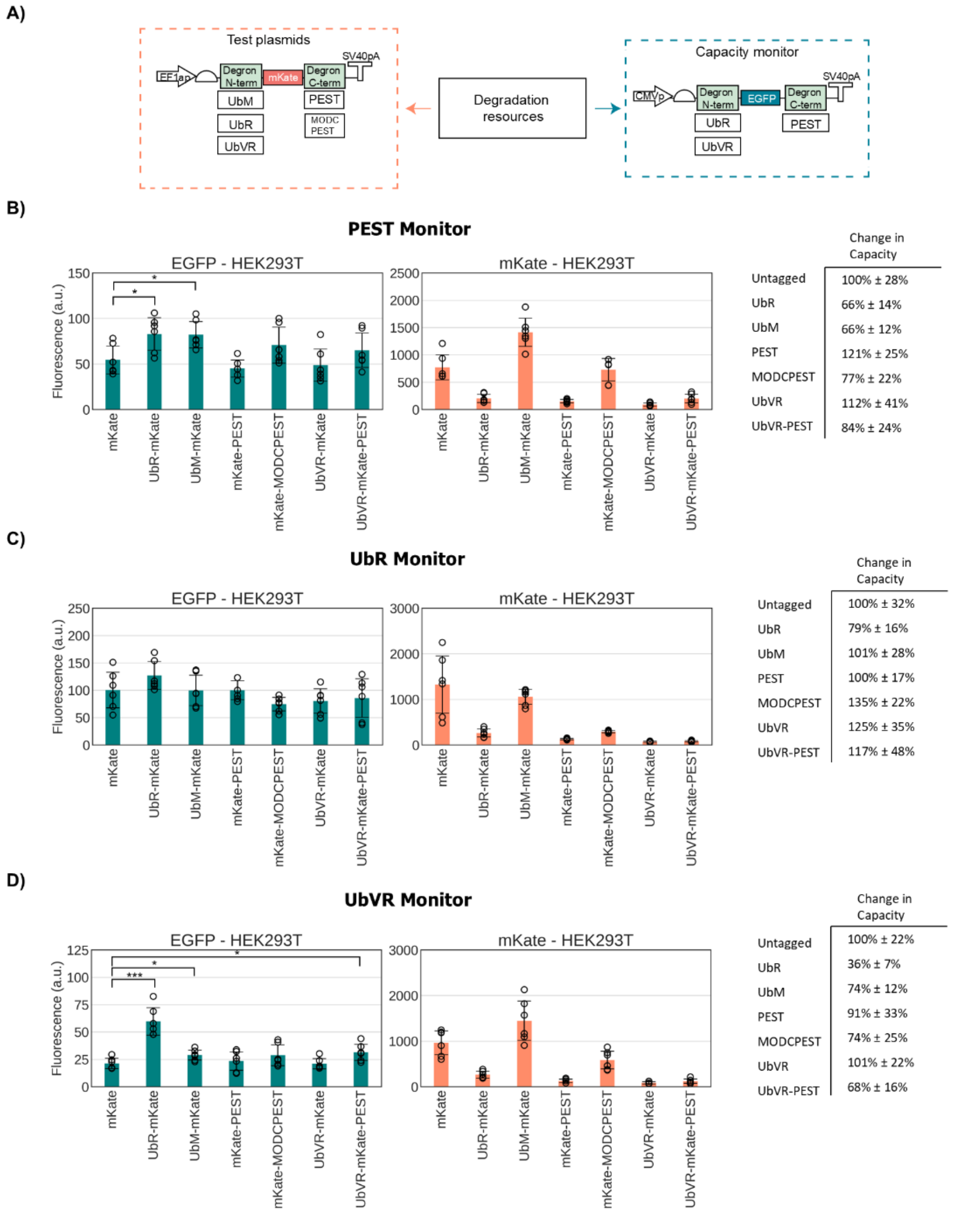

Figure S2. Characterisation of a library of degrons with three co-transfected EGFP monitors in HEK293T cells. A) Schematics of the genetic construct library considered where three EGFP-expressing monitors tagged with UbR, UbVR and PEST respectively were co-transfected with six mKate-expressing test constructs tagged with UbM, UbR, UbVR, PEST or MODCPEST. **B)** Flow cytometry data for the EGFP monitor with PEST. **C)** Flow cytometry data for the EGFP monitor with UbR. **D)** Flow cytometry data for the EGFP monitor with UbVR. 2xUbAV-mKate is missing due to a repeated failure in DNA assembly of the construct. Test plasmid and capacity monitor fluorescence levels are reported as mean fluorescence (arbitrary units) ± std. Data derived from three independent experiments each with two biological repeats. A two-tailed Student T-Test was used, where P values are denoted as follows: *****<0.00005, ****<0.0005, ***<0.0005, **<0.005, *<0.05, and it was annotated only on the mKate charts with reference to the control, EGFP versus capacity monitor, for clarity purposes. Tables report 1/(EGFP of mKate-tagged condition/EGFP of mKate untagged condition) to quantify change in degradation capacity upon adding the degron ± the ratio of the standard deviation to the EGFP mean multiplied by change in capacity. The number of biological repeats for each sample and exact P values are reported in Source data file.

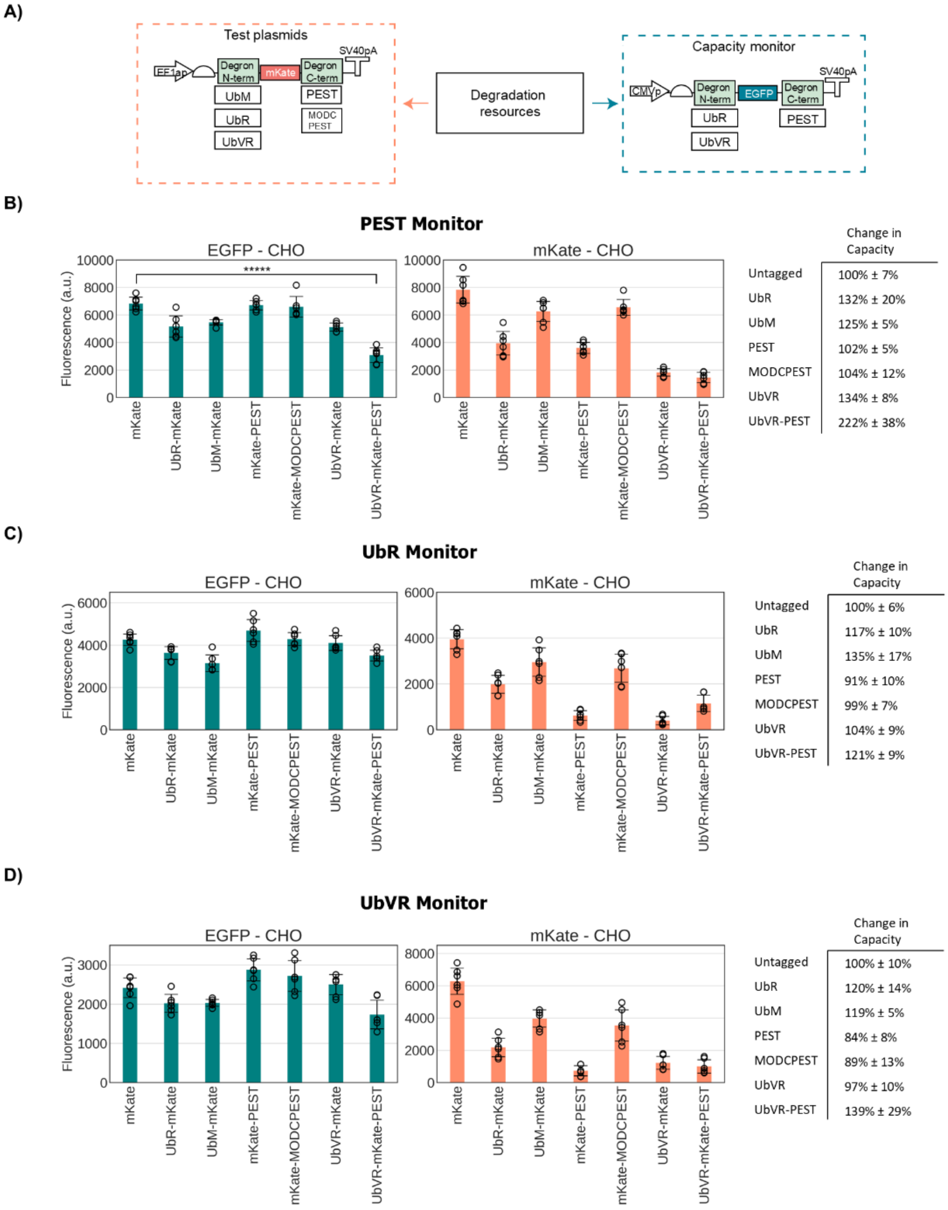

Figure S3. Characterisation of a library of degrons with three co-transfected EGFP monitors in CHO-K1 cells. A) Schematics of the genetic construct library considered where three EGFP-expressing monitors tagged with UbR, UbVR and PEST respectively were co-transfected with six mKate-expressing test constructs tagged with UbM, UbR, UbVR, PEST or MODCPEST. **B)** Flow cytometry data for the EGFP monitor with PEST. **C)** Flow cytometry data for the EGFP monitor with UbR. **D)** Flow cytometry data for the EGFP monitor with UbVR. 2xUbAV-mKate is missing due to a repeated failure in DNA assembly of the construct. Test plasmid and capacity monitor fluorescence levels are reported as mean fluorescence (arbitrary units) ± std. Data derived from three independent experiments each with two biological repeats. A two-tailed Student T-Test was used, where P values are denoted as follows: *****<0.00005, ****<0.0005, ***<0.0005, **<0.005, *<0.05, and it was annotated only on the mKate charts with reference to the control, EGFP versus capacity monitor, for clarity purposes. Tables report 1/(EGFP of mKate-tagged condition/EGFP of mKate untagged condition) to quantify change in degradation capacity upon adding the degron ± the ratio of the standard deviation to the EGFP mean multiplied by change in capacity. The number of biological repeats for each sample and exact P values are reported in Source data file.

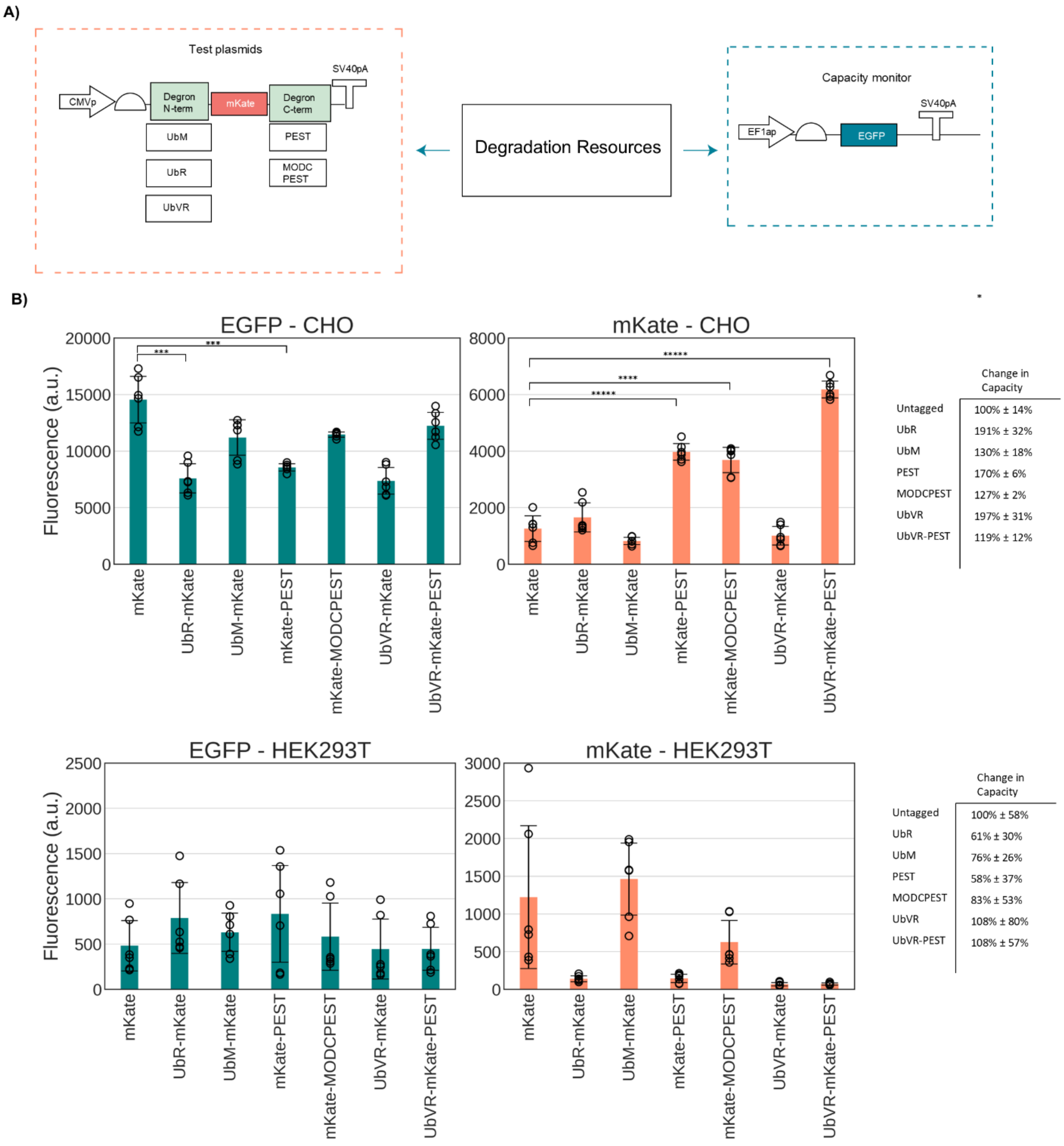

Figure S4. An untagged EGFP monitor results in the absence of competition for degradation. A) Schematics of the genetic construct library where the untagged EGFP monitor was co-transfected with six mKate-expressing test constructs tagged with UbM, UbR, UbVR, PEST or MODCPEST in both HEK293T and CHO-K1. **B)** Flow cytometry data for the untagged monitor in CHO-K1 (top) and HEK293T (bottom). 2xUbAV-mKate is missing due to a repeated failure in DNA assembly of the construct. Test plasmid and capacity monitor fluorescence levels are reported as mean fluorescence (arbitrary units) ± std. Data derived from three independent experiments each with two biological repeats. A two-tailed Student T-Test was used, where P values are denoted as follows: *****<0.00005, ****<0.0005, ***<0.0005, **<0.005, *<0.05, and it was annotated only on the mKate charts with reference to the control, EGFP versus capacity monitor, for clarity purposes. Tables report 1/(EGFP of mKate-tagged condition/EGFP of mKate untagged condition) to quantify change in degradation capacity upon adding the degron ± the ratio of the standard deviation to the EGFP mean multiplied by change in capacity. The number of biological repeats for each sample and exact P values are reported in Source data file.

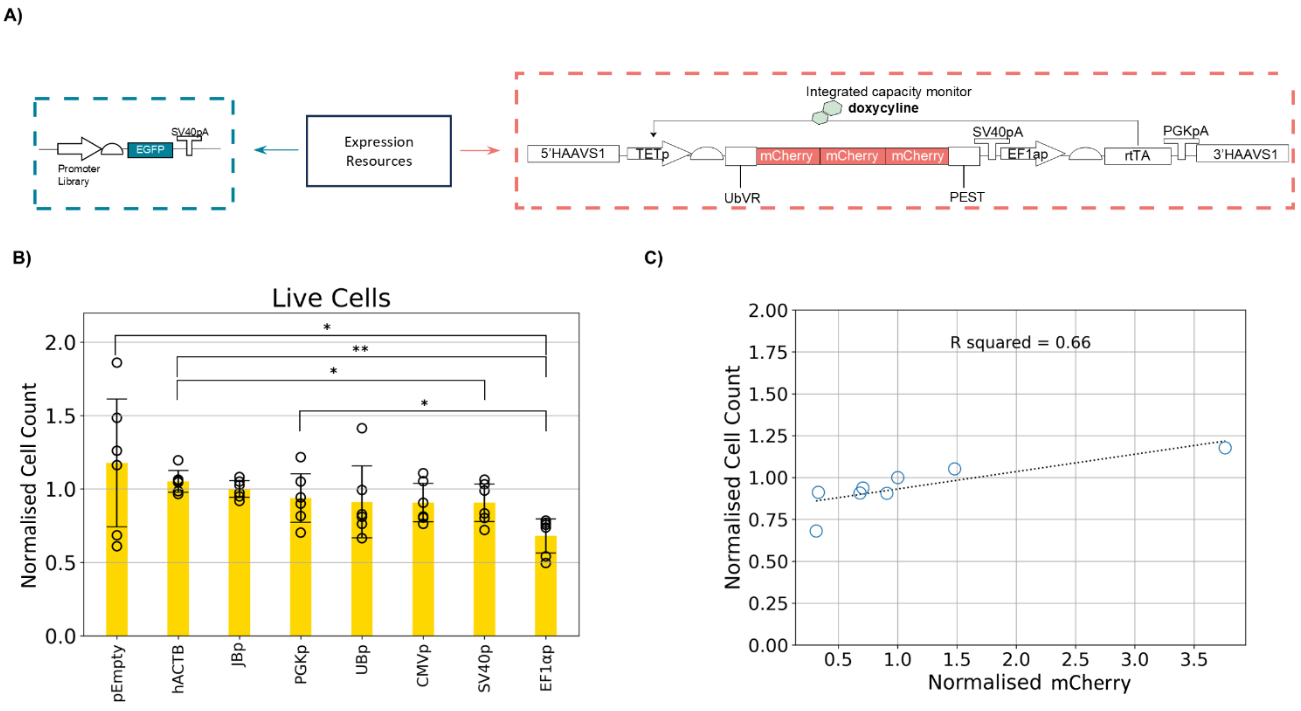

Figure S5. **Effects of competition for gene expression resources on cell growth. A)** A library of test plasmids bearing different promoters was selected to test the monitor response to competition. **B)** Cell count data after two days of expression of the test plasmid versus the induced integrated capacity monitor. In these analyses, cell counts are reported as normalized mean cell count (fold-change) ±standard deviation. **C)** Scatter plot of normalised mKate and normalised cell count with linear regression represented as a dotted line and annotated R^2. The data presented are derived from three independent experiments each comprised of two biological repeats. Statistical significance was determined using a two-tailed Student T-Test, where P values are denoted as follows: *****<0.00005, ****<0.0005, ***<0.0005, **<0.005, *<0.05. The number of biological repeats for each sample, the normalization, and exact P values are reported in the Source Data File.

Supplementary Note 1

Transfection Flow Cytometry Data Gating Strategies in the Context Resource Competition

Resource competition studies are performed via transfection/transformation of one plasmid into a cell carrying a "capacity monitor", a fluorescent reporter resource-coupled to the plasmid, or multiple plasmids when the "capacity monitor" is not genomically integrated. The analysis of transfection data is usually either carried in bulk via fluorescence or luminescence in a plate reader, via mRNA levels with qPCR and RNAseq or at the single cell level with flow cytometry or imaging techniques. In our article, we adopted flow cytometry given its sensibility and the granularity of the output. Flow cytometry allows to gate populations of cells, excluding ones that might not be of interest for the analysis, or might confound it, such as debris, dead cells, doublets, untransfected. Outlined here is the choice of our gating strategy:

1. Gating out debris and dead cells via an FSC/SSC gate.
2. Gating out of doublets via an FSC-A/FSC-H gate.
3. Statistical computation of geometric mean fluorescence of gated cells.

This gating strategy tends to underestimate competition effects and introduces variability due to the unpredictable variance in transfection efficiency. We consider the strategy to be high accuracy but low precision. However, alternative strategies based on gating fluorescent markers are high precision but risk low accuracy in a resource competition setting.

Gating strategies based on fluorescent markers employ either a gate on a transfection marker, a third transfected fluorescent protein, or on a transfected fluorescent protein which is part of the experiment. Both strategies are subject to competition for expression resources by the other expression cassettes, thereby inducing a condition-dependent bias in the data:

- If the transfection marker is part of an experiment with high competition for expression resources, it might be suppressed to a level where the cell population can be mistaken for untransfected cells.
- In another possible instance, fluorescent proteins being measured as part of the experiment are being used to gate out untransfected cells, however, in different conditions, different competing constructs, their levels might drastically increase or decrease, over and underestimating the results on a condition-by-condition basis.

In competition for expression resources, we might see underestimation of the transfected population in conditions of high competition as these will lower expression levels of the monitor, while in competition for degradation experiments the opposite will happen as higher degradation competition leads to an increase in undegraded, therefore fluorescent, monitor levels.

Methods derived from gating on a transfection marker or on a fluorescent protein part of the experiment, such as gating on a fixed percentile of the markers are still affected by resource competition effects and we deem them high precision, low accuracy.

We show how gating for fluorescent transfected proteins to remove untransfected cells can mislead towards the false identification of a significant difference between the capacity monitor competing with an induced degron (Figure S6A). The histograms overlaid to the flow cytometry dot plot highlight the difference between uninduced and induced ecDHFR degron only in the EGFP channel, ~10-fold decrease (Figure S6B, C). However, in the bar plots representing the geometric mean of the gated version of the data a ~2-fold difference appears in the mKate between the uninduced and induced condition which is not reflected in the raw data (Figure S6C).

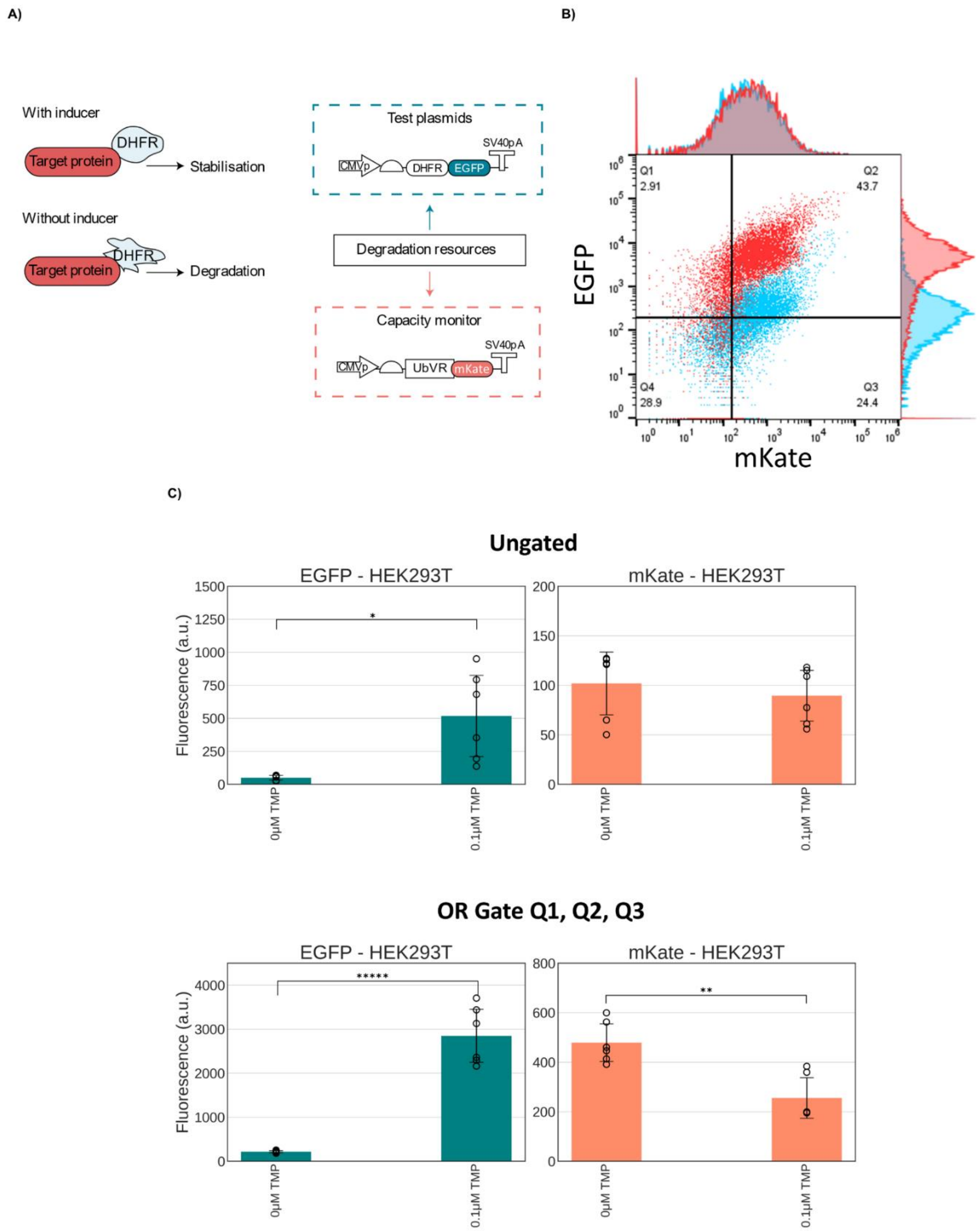

Figure S6. Different flow cytometry gating strategies and their effect on the analysis of resource competition data. A) A diagram of the representative experiment on which we tested the two different gating strategies. **B)** A dot plot with overlaid histograms displaying in blue an uninduced ecDHFR-EGFP competing with a capacity monitor UbVR-mKate, and in red the induced version with 0.1µM of TMP. **C)** Bar plots representing the geometric mean of flow cytometry data for EGFP and mKate. In these analyses, test plasmid and capacity monitor intracellular protein levels are reported as mean fluorescence (arbitrary units) ± standard deviation. The data presented are derived from three independent experiments each comprised of two biological repeats. Statistical significance was determined using a two-tailed Student T-Test, where P values are denoted as follows: *****<0.00005, ****<0.0005, ***<0.0005, **<0.005, *<0.05. The number of biological repeats for each sample and exact P values are reported in the Source Data File.

Supplementary Note 2

Generation of the integrated capacity monitor

The all-in-one plasmid used for the integration of our capacity monitor, pRD28 detailed in Supplementary data file 1 and whose map is in Supplementary data file 2) was built from the hCas9 plasmid from the Church lab (https://www.addgene.org/41815/). The guide RNA was:
5'-GGGGCCACTAGGGACAGGAT-3'
Which was validated in Yang, L. *et al.* 2013.
The homology regions for the homology directed repair in the AAVS1 locus were at the 5' end of the cargo:
5'-
tagggcgatcgctgctttctctgaccagcattctctcccctgggcctgtgccgctttctgtctgcagcttgtggcctgggtcacctctacggctggcccagatccttc
cctgccgcctccttcaggttccgtcttcctccactccctcttccccttgctctctgctgtgttgctgcccaaggatgctctttccggagcacttccttctcggcgctgca
ccacgtgatgtcctctgagcggatcctccccgtgtctgggtcctctccgggcatctctcctccctcacccaaccccatgccgtcttcactcgctgggttcccttttcc
ttctccttctggggcctgtgccatctctcgtttcttaggatggccttctccgacggatgtctcccttgcgtcccgcctccccttcttgtaggcctgcatcatcaccgttttt
ctggacaaccccaaagtaccccgtcttcctggctttagccacctctccatcctcttgctttctttgcctggacaccccgttctcctgtggattcgggtcacctctcact
cctttcatttgggcagctcccctaccccccttacctctctagtctgtgctagctcttccagccccctgtcatggcatcttccaggggtccgagagctcagctagtctt
cttcctccaacccgggcccctatgtccacttcaggacagcatgtttgctgcctccagggatcctgtgtccccgagctgggaccaccttatattcccagggccgg
ttaatgtggctctggttctgggtacttttatctgtcccctccaccccacagtggggcggtacc-3'
and:
5'-
cacgacgcgtactagggacaggattggtgacagaaaagccccatccttaggcctcctccttcctagtctcctgatattgggtctaacccccacctcctgttagg
cagattccttatctggtgacacacccccatttcctggagccatctctctccttgccagaacctctaaggtttgcttacgatggagccagagaggatcctgggag
ggagagcttggcaggggggtgggagggaagggggggatgcgtgacctgcccggttctcagtggccaccctgcgctaccctctcccagaacctgagctgct
ctgacgcggctgtctggtgcgtttcactgatcctggtgctgcagcttccttacacttcccaagaggagaagcagtttggaaaaacaaaatcagaataagttggt
cctgagttctaactttggctcttcacctttctagtccccaatttatattgttcctccgtgcgtcagttttacctgtgagataaggccagtagccagccccgtcctggca
gggctgtggtgaggaggggggtgtccgtgtggaaaactccctttgtgagaatggtgcgtcctaggtgttcaccaggtcgtggccgcctctactccctttctctttc
tccatccttctttccttaaagagtccccagtgctatctgggacatattcctccgcccagagcagggtcccgcttccctaaggccctgctctgggcttctgggtttga
gtccttggcaagcccaggagaggcgctcaggcttccctgtccccccttcctcgtccaccatctcatgcccctggctctcctgccccttccctacaggggttcctgg
ctctgctctgcggccgc-3'
At the 3' end of the cargo.

The primers used for validation of successful knock-in of our capacity monitor were:

Primer ID	Orientation	Sequence	Notes
ID104	Fwd	CTGCCGTCTCTCTCCTGAGT	Anneals to the AAVS1 locus before the integrated cargo.
ID144	Rev	CGCAATTAATTCTGACGGTTCACTAA	Anneals on a connector sequence within the integrated payload.
ID301	Rev	CGAGGGTAGGAAGTGGTACGG	Anneals in the TreGp promoter within the integrated cargo.
ID122	Rev	AATCTGCCTAACAGGAGGTGG	Anneals in the AAVS1 3' homology region.

Supplementary Note 3

List of plasmids adopted in this study

**Degron test plasmids and Capacity Monitors**
**Expression test plasmids**
**Intermediate Assembly Vectors**
**Cas9 all-in-one plasmid**

Color code	Plasmid Name	Plasmid composition
Degron test plasmids and Capacity Monitors (light green)	JR06	CMVp-mKate-PEST-SV40pA
	JR07	CMVp-UbR-mKate-SV40pA
	JR08	CMVp-UbVR-mKate-SV40pA
	pRD153	TreGp-UbVR-3xmCherry-PEST-SV40pA
	JR01	EF1ap-UbR-EGFP-SV40pA
	JR02	EF1ap-UbM-EGFP-SV40pA
	JR03	EF1ap-2xUbAV-EGFP-SV40pA
	JR04	EF1ap-PEST-EGFP-SV40pA
	JR05	EF1ap-MODCPEST-EGFP-SV40pA
	pSC-DD1	CMVp-ecDHFR-EGFP-SV40pA
	pAY15	CMVp-osTIR(F74G)-P2A-EGFP-SV40pA
	JR23	CMVp-UbM-mKate-SV40pA
	JR25	CMVp-mKate-MODCPEST-SV40pA
	JR26	CMVp-UbVR-mKate-PEST-SV40pA
Expression test plasmids (medium green)	pRD112	pEmpty
	pRD113	hACTBp-Kz1-EGFP-SV40pA
	pRD114	pJB42CAT5-Kz1-EGFP-SV40pA
	pRD115	SV40p-Kz1-EGFP-SV40pA
	pRD116	EF1ap-Kz1-EGFP-SV40pA
	pRD144	CMVp-Kz1-EGFP-SV40pA
	pRD177	PGKp-Kz1-EGFP-SV40pA
	pRD182	UBp-Kz1-EGFP-SV40pA
Intermediate Assembly Vectors (green)	JR19	CMVp-UbM-EGFP-SV40pA
	JR21	CMVp-EGFP-MODCPEST-SV40pA
	JR22	CMVp-UbVR-EGFP-PEST-SV40pA
Cas9 all-in-one plasmid (dark green)	pRD28	CMVp-Cas9-T2A-mKate-hPEST-SV40pA-U6-AAVS1gRNA

Supplementary Data Files provided with this study

Supplementary Data 1. List of primers and their sequences used in this study.

Supplementary Data 2. Details on the transfection reactions performed in the described experiments.
