# Supplementary material for: Degradation bottlenecks and resource competition in transiently and stably engineered mammalian cells
Source: Nat Commun. 2025 Jan 2;16:328. doi: 10.1038/s41467-024-55311-w (PMC11696530; doi:10.1038/s41467-024-55311-w)
Supplement: Supplementary file 2 — Description of Additional Supplementary Files [file 41467_2024_55311_MOESM2_ESM.pdf]

Legend for Supplementary Data Files

Supplementary Data 1. List of primers and their sequences used in this study

Supplementary Data 2. Details on the transfection reactions performed in the described experiments
